# Cardiac Involvement in Recovered Patients From COVID-19: A Preliminary 6-Month Follow-Up Study

**DOI:** 10.3389/fcvm.2021.654405

**Published:** 2021-05-13

**Authors:** Xiaoyan Wu, Ke-Qiong Deng, Chenze Li, Zhaoxia Yang, Huijuan Hu, Huanhuan Cai, Chao Zhang, Tao He, Fang Zheng, Hairong Wang, Xin A. Zhang, Antoine Caillon, Yufeng Yuan, Xinghuan Wang, Haibo Xu, Zhibing Lu

**Affiliations:** ^1^Department of Cardiology, Zhongnan Hospital of Wuhan University, Wuhan, China; ^2^Department of Radiology, Zhongnan Hospital of Wuhan University, Wuhan, China; ^3^Department of Clinical Laboratory, Zhongnan Hospital of Wuhan University, Wuhan, China; ^4^University of Oklahoma Health Science Center, Oklahoma City, OK, United States; ^5^Hypertension and Vascular Research Unit, Lady Davis Institute for Medical Research, Sir Mortimer B. Davis-Jewish General Hospital, McGill University, Montreal, QC, Canada; ^6^Department of Hepatobiliary and Pancreatic Surgery, Zhongnan Hospital of Wuhan University, Wuhan, China; ^7^Center for Evidence-Based and Translational Medicine, Zhongnan Hospital of Wuhan University, Wuhan, China

**Keywords:** cardiac magnetic resonance imaging, fibrosis, follow-up, cardiac injury, COVID-19

## Abstract

**Background:** Accumulating evidence has revealed that coronavirus disease 2019 (COVID-19) patients may be complicated with myocardial injury during hospitalization. However, data regarding persistent cardiac involvement in patients who recovered from COVID-19 are limited. Our goal is to further explore the sustained impact of COVID-19 during follow-up, focusing on the cardiac involvement in the recovered patients.

**Methods:** In this prospective observational follow-up study, we enrolled a total of 40 COVID-19 patients (20 with and 20 without cardiac injury during hospitalization) who were discharged from Zhongnan Hospital of Wuhan University for more than 6 months, and 27 patients (13 with and 14 without cardiac injury during hospitalization) were finally included in the analysis. Clinical information including self-reported symptoms, medications, laboratory findings, Short Form 36-item scores, 6-min walk test, clinical events, electrocardiogram assessment, echocardiography measurement, and cardiac magnetic resonance imaging was collected and analyzed.

**Results:** Among 27 patients finally included, none of patients reported any obvious cardiopulmonary symptoms at the 6-month follow-up. There were no statistically significant differences in terms of the quality of life and exercise capacity between the patients with and without cardiac injury. No significant abnormalities were detected in electrocardiogram manifestations in both groups, except for nonspecific ST-T changes, premature beats, sinus tachycardia/bradycardia, PR interval prolongation, and bundle-branch block. All patients showed normal cardiac structure and function, without any statistical differences between patients with and without cardiac injury by echocardiography. Compared with patients without cardiac injury, patients with cardiac injury exhibited a significantly higher positive proportion in late gadolinium enhancement sequences [7/13 (53.8%) vs. 1/14 (7.1%), *p* = 0.013], accompanied by the elevation of circulating ST2 level [median (interquartile range) = 16.6 (12.1, 22.5) vs. 12.5 (9.5, 16.7); *p* = 0.044]. Patients with cardiac injury presented higher levels of aspartate aminotransferase, creatinine, high-sensitivity troponin I, lactate dehydrogenase, and N-terminal pro–B-type natriuretic peptide than those without cardiac injury, although these indexes were within the normal range for all recovered patients at the 6-month follow-up. Among patients with cardiac injury, patients with positive late gadolinium enhancement presented higher cardiac biomarker (high-sensitivity troponin I) and inflammatory factor (high-sensitivity C-reactive protein) on admission than the late gadolinium enhancement–negative subgroup.

**Conclusions:** Our preliminary 6-month follow-up study with a limited number of patients revealed persistent cardiac involvement in 29.6% (8/27) of recovered patients from COVID-19 after discharge. Patients with cardiac injury during hospitalization were more prone to develop cardiac fibrosis during their recovery. Among patients with cardiac injury, those with relatively higher cardiac biomarkers and inflammatory factors on admission appeared more likely to have cardiac involvement in the convalescence phase.

## Introduction

The novel coronavirus disease 2019 (COVID-19) caused by severe acute respiratory syndrome coronavirus 2 (SARS-CoV-2) infection has developed into an unprecedented global pandemic (1). To date, more than 140 million confirmed cases have been identified in more than 200 countries around the world, according to the latest data from the World Health Organization. The clinical presentation of COVID-19 is mostly characterized by respiratory symptoms, and the lung is the major organ involved, causing complications related to pneumonia and acute respiratory distress syndrome (2). Accumulating evidence has revealed that COVID-19 affects multiple organs including the cardiovascular system (3–6). In our previous report, we have demonstrated that myocardial injury with troponin elevation is significantly associated with fatal outcomes of COVID-19 patients, which has been confirmed by other studies (7–12). However, it is unknown whether patients with cardiac injury during hospitalization suffer from a sustained myocardial impairment, cardiac sequelae during their convalescence, and the implications of persistent cardiac involvement on the consequence are not clear.

Recent follow-up studies have demonstrated that patients in the early convalescence may suffer from impairment of pulmonary function, symptoms of fatigue, and physical and psychological damage after their discharge for 1 month (13–16). Some studies have also reported short-term cardiac involvement in convalescent patients (17, 18). Nevertheless, longer follow-up studies are needed to fully evaluate the long-term cardiac impact of COVID-19. In this study, the patients with COVID-19 who were discharged from our hospital for more than 6 months were enrolled and analyzed. The purpose of our study was to further observe the persistent impact of COVID-19 during follow-up, focusing on the cardiac involvement in the recovered patients.

## Methods

### Study Design and Participants

A prospective observational study was designed to investigate the long-term prognosis of COVID-19 patients with or without cardiac injury during hospitalization. The study protocol was approved by the ethics committees of Zhongnan Hospital of Wuhan University and conducted in accordance with the Declaration of Helsinki and the International Conference on Harmonization Guidelines for Good Clinical Practice. Written informed consent was obtained from all participants.

The participants were consecutively recruited at Zhongnan Hospital of Wuhan University from March 1 to April 1, 2020. Initially, 32 patients with cardiac injury during hospitalization were screened for eligibility. The enrolled patients had to meet the following eligibility requirements: (1) patients were older than 18 years old; (2) patients were diagnosed as COVID-19 based on a positive real-time polymerase chain reaction (RT-PCR) for SARS-CoV-2 in association with clinical symptoms according to the diagnosis and treatment guideline published by the National Health Commission of China during hospitalization; (3) patients had cardiac injury during hospitalization. Cardiac troponin I (cTnI) was measured using a high-sensitivity cTnI (hs-cTnI) assay (Abbott ARCHITECT). The assay's limit of detection is 1.9 pg/mL, the 99th percentile upper reference limit (URL) is 26.2 pg/mL, and the coefficient of variation at 26.2 pg/mL is <5%. Cardiac injury was defined as at least one cTn concentration is above the 99th percentile URL (>26.2 pg/mL); (4) patients who recovered from SARS-CoV-2 infection and discharged from the hospital for more than 6 months at follow-up; (5) patients consented to participate in the follow-up study. Patients were discharged if a combination of the following criteria was satisfied according to the guideline of the Chinese Center for Disease Control and Prevention: the absence of fever for at least 3 days, clinical remission of respiratory symptoms, substantial improvement in acute exudative lesions on chest computed tomography scan, and two consecutive throat swabs negative RT-PCR test results for SARS-CoV-2 RNA obtained at least 24 h apart. The baseline clinical information, including demographic characteristics, coexisting diseases, laboratory findings, and clinical treatments during hospitalization, was collected using a standardized case report form at the time of patients' enrollment via electronic medical charts. Besides, the patients with the following conditions were excluded from this study: (1) patients had contraindications to the examination of cardiac magnetic resonance (CMR) imaging; (2) patients had a history of coronary heart disease or cardiomyopathy before admission; (3) patients had a malignant tumor for which life expectancy was <6 months; (4) liver or kidney dysfunction unrelated to COVID-19 [aspartate aminotransferase (AST) or alanine aminotransferase (ALT) is 2 times higher than the upper threshold; creatinine >90 μmol/L]. Finally, 20 patients with cardiac injury were included in the schedule for routine follow-up appointments in the outpatient clinic 6 months after discharge. For comparisons, 20 age- and gender-matched COVID-19 patients without cardiac injury were included as controls in this prospective study. Their inclusion and exclusion criteria were consistent with that in patients with cardiac injury, except for the requirements of elevated hs-cTnI level during hospitalization.

### The Clinical Follow-Up

Investigators were instructed to contact patients and perform face-to-face interviews at predesignated times after discharge, to collect relevant clinical information. The information included self-reported symptoms, medications, laboratory findings (biomarkers for liver and renal function, coagulation, inflammation, and myocardial injury), quality-of-life scores [Short Form 36-item questionnaire (SF-36)], 6-min walk test, clinical events, electrocardiogram (ECG), echocardiography, and CMR imaging. Also, the serum level of soluble ST2 was quantified using a commercial enzyme-linked immunosorbent assay kit (R&D Systems, DST200). The observation window of follow-up was defined as the interval range from the date of discharge to the date of the last contact. If a patient was only contacted by physicians via telephone follow-up and refused to undergo laboratory and imaging examinations, the patient was then not included in the analysis. The last follow-up date was October 17, 2020.

### Quality-of-Life Assessment

The quality of life of the patients at follow-up was evaluated by the SF-36 survey. The SF-36 scale was composed of 36 items, which can be divided into eight dimensions, including physical functioning, role limitation due to physical problems (role physical), bodily pain, general health, vitality, social functioning, and role limitation due to emotional health problems (role emotional). Each dimension was scored separately from 0 to 100, with high values representing better functional status.

### Exercise Tolerance Test

Exercise endurance of patients was performed by a 6-min walk test without supplemental oxygen. Measurements of heart rate (HR), systolic/diastolic blood pressure, and percutaneous oxygen saturation (Spo_2_) were measured. Rating of perceived exertion with Borg scale in patients was also scored after the 6-min walk test.

### Transthoracic Echocardiography

The cardiac structure and function of patients were evaluated by sequential transthoracic echocardiography scans using a P4-2S ultrasound scanner (Mindray; Shenzhen, China). From the parasternal long-axis view, we obtained the systolic and diastolic measurements, including left atrial (LA) dimension, left ventricular (LV) dimension, right atrial (RA) dimension, right ventricular (RV) dimension, interventricular septum thickness (IVS), and LV posterior wall thickness (LVPW). Tricuspid annular plane systolic excursion (TAPSE) was measured as the systolic displacement of the tricuspid lateral annulus on M-mode imaging. We used M-mode echocardiography to calculate LV ejection fraction (LVEF).

### CMR Imaging

A 3.0-T MR scanner (Prisma, Siemens Healthcare, Germany) was applied to obtain CMR imaging in all patients. The data were collected through an 18-channel phased-array body coil combined with 12 channels from the spine coil. Patients with a HR higher than 75 beats/min were administered with β-blocker (metoprolol, 25–50 mg). We first used the conventional CMR scan protocol, including long- and short-axis cine and late gadolinium enhancement (LGE) to obtain images, and then native T1 mapping and extracellular volume (ECV) were quantitatively evaluated. T1 myocardial mapping was collected in three locations covering the base, midventricle, and apex of the short-axis LV by a modified Look-Locker inversion recovery pulse sequence before contrast administration. After patients were administered with 0.10–0.15/kg gadoterate meglumine (Dotarem; Guerbet AG, Paris, France), LGE sequences were obtained approximately for 10–15 min. Postcontrast T1 mapping was obtained approximately for 15–20 min after gadoterate meglumine administration, and postcontrast T1 mapping was collected using the same imaging plane as the pre-contrast T1 mapping. Two experienced radiologists blindly analyzed all CMR images using a commercial software cvi 42, v.5.3 (Circle Cardiovascular Imaging, Calgary, Alberta, Canada). If there are any discrepancies between the two radiologists, another senior radiologist adjudicated the CMR imaging.

### Statistical Analysis

Patients were divided into two groups based on hs-cTnI level throughout hospitalization, and their baseline characteristics and follow-up findings were then compared. The continuous data were presented as median [interquartile range (IQR)], and their comparisons between groups were performed by Mann–Whitney *U* tests. For categorical variables, data were expressed as frequency (percentage) and compared by Fisher exact test. R version 3.4.0 (Vienna, Austria) was used to perform statistical analysis. All comparisons were two-sided, and a *p* < 0.05 was considered statistically significant.

## Results

### Patient Characteristics

**Figure 1:**
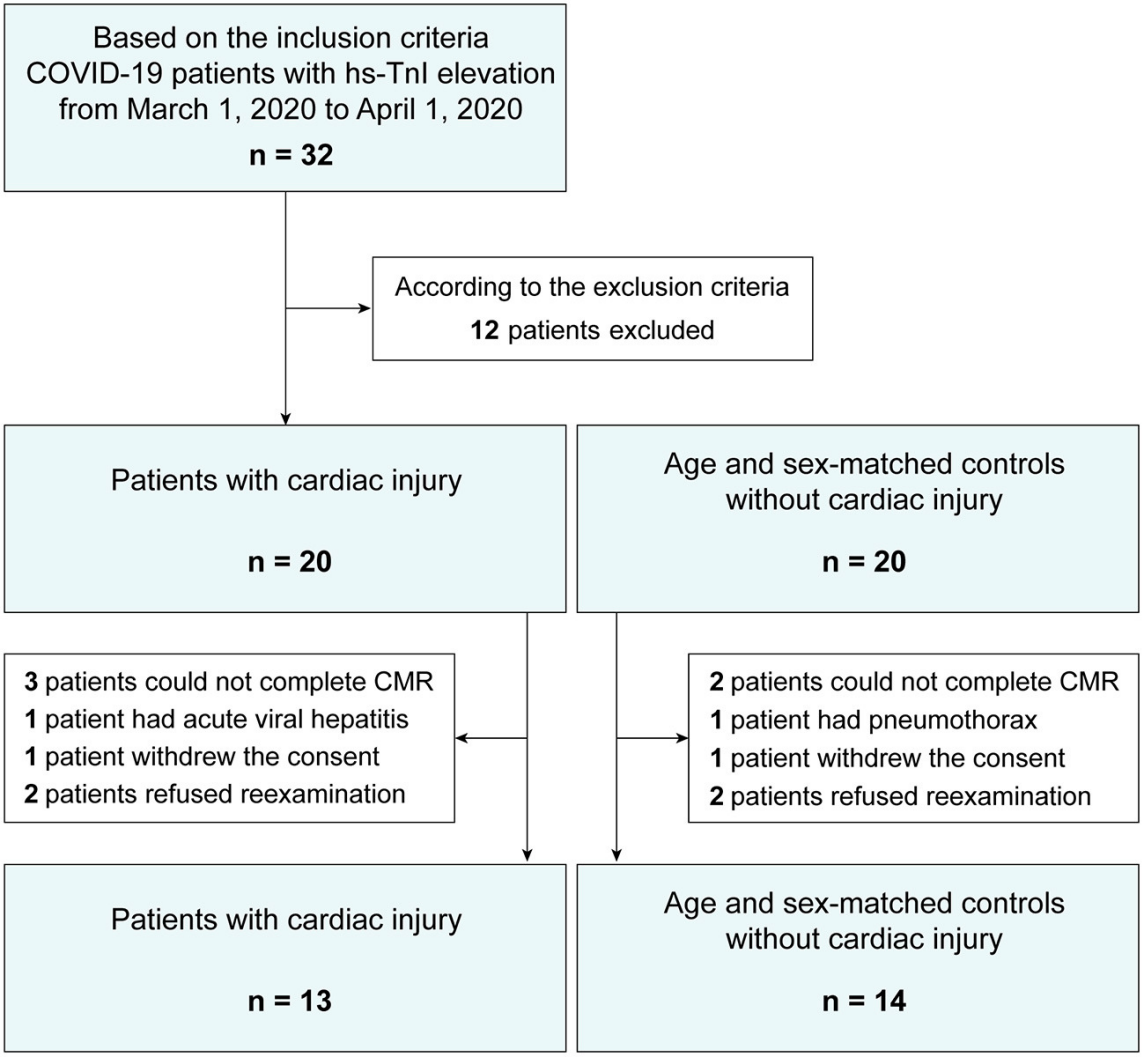
A flowchart of patient recruitment.

A flowchart for patient recruitment is illustrated in Figure 1. Based on the inclusion and exclusion criteria, 40 consecutive patients admitted to our hospital from March 1 to April 1, 2020, were initially followed up. No patients were readmitted for cardiopulmonary reasons or died during follow-up. Five patients could not complete the examination of CMR for allergic reaction to contrast media (two patients), claustrophobia (one patient), unwillingness (one patient), and presence of metal implants during follow-up (one patient). One patient had severe liver dysfunction for acute viral hepatitis. One patient suffered from pneumothorax caused by thoracic trauma. Two patients dropped out of the study because of withdrawal of consent. Four patients refused to return to the hospital for reexamination because of fearing reinfection. At last, a total of 27 patients who recovered from COVID-19 for at least 6 months were enrolled for analysis, of which myocardial injury with positive troponin (hs-TnI >26.2 pg/mL) throughout hospitalization was confirmed in 13 patients (exclusion of acute coronary syndrome), and 14 age- and gender-matched patients without cardiac injury were included as controls.

The details of baseline characteristics are presented in Table 1. The median ages were 63 years (IQR = 59–70 years) in patients with cardiac injury and 63 years (IQR = 57–70 years) in those without cardiac injury, respectively. Of the 27 patients, 16 were diagnosed with moderate-type COVID-19, 8 with severe-type, and 3 with critical type, according to the Diagnosis and Treatment Protocol of Novel Coronavirus issued by the National Health Commission of the People's Republic of China (fifth version). Patients with cardiac injury were tended to be identified with more severe and critical types (8/13 vs. 3/14; Table 1). The median duration of hospital stay was 11 days (IQR = 8–18 days). Comorbidities were presented in nine patients, with a history of diabetes in five patients (18.5%) and hypertension in four patients (14.8%). During COVID-19 hospitalization, 81.5% of patients underwent antiviral therapy (19/27), antibiotics (12/27), corticosteroids (5/27), immunoglobulin (1/27), oxygen inhalation (16/27), and mechanical ventilation (2/27). Angiotensin-converting enzyme inhibitor (1/27), β-blocker (2/27), calcium-channel blocker (4/27), and statin (2/27) were applied in these patients after discharge. There were no statistical differences in age, gender, underlying commodities, therapeutic history, and medication after discharge between the patients with and without cardiac injury. For the laboratory values on admission, patients with cardiac injury showed significantly higher levels of lactate dehydrogenase (LDH), creatinine, and N-terminal pro–B-type natriuretic peptide (NT-proBNP) compared with those without (all *p* < 0.05). However, the elevation of hs-cTnI was marginally significant (*p* = 0.061) on admission, although hs-cTnI levels were confirmed to be significantly elevated in all patients with cardiac injury during their hospitalization.

**Table 1:** Baseline characteristics of study subjects.

**Characteristics**	**Overall**	**Patients with cardiac injury**	**Patients without cardiac injury**	***p***
	**(*n* = 27)**	**(*n* = 13)**	**(*n* = 14)**	
Age (years)	63 [58, 70]	63 [59, 70]	63 [57, 70]	0.697
Male *n* (%)	8 (29.6)	4 (30.8)	4 (28.6)	0.999
Illness classification *n* (%)				0.054
Mild	16 (59.3)	5 (38.5)	11 (78.6)	
Severe/critical	11 (40.7)	8 (61.5)	3 (21.4)	
Length of hospital stay (days)	11 [8, 18]	11 [9, 24]	10 [8, 13]	0.205
**Presence of comorbidities**				
History of hypertension *n* (%)	4 (14.8)	1 (7.7)	3 (21.4)	0.596
History of diabetes mellitus *n* (%)	5 (18.5)	2 (15.4)	3 (21.4)	0.999
History of coronary heart disease *n* (%)	0 (0.0)	0 (0.0)	0 (0.0)	–
History of heart failure *n* (%)	0 (0.0)	0 (0.0)	0 (0.0)	–
History of atrial fibrillation *n* (%)	0 (0.0)	0 (0.0)	0 (0.0)	–
History of cardiomyopathy *n* (%)	0 (0.0)	0 (0.0)	0 (0.0)	–
**Laboratory values on admission**				
WBC (10^9^/L)	5.7 [4.6, 6.7]	5.9 [4.5, 8.1]	5.5 [4.7, 6.6]	0.607
Hb (10^12^/L)	121 [114, 130]	123 [112, 129]	120 [115, 130]	0.797
PLT (10^9^/L)	203 [169, 253]	192 [178, 238]	216 [165, 253]	0.738
ALT (U/L)	32 [20, 45]	32 [24, 42]	28 [17, 45]	0.425
AST (U/L)	27 [19, 35]	32 [24, 48]	22 [16, 30]	0.057
LDH (U/L)	258 [171, 338]	332 [227, 422]	185 [156, 274]	0.046
Creatinine (umol/L)	52 [46, 67]	64 [51, 76]	51 [44, 59]	0.027
hs-cTnI (pg/mL)	6.3 [2.2, 14.6]	9.9 [5.6, 20.6]	5.3 [2.0, 6.8]	0.061
NT-proBNP (pg/mL)	278 [121, 388]	390 [324, 530]	123 [116, 274]	0.020
hs-CRP (mg/L)	10.8 [2.6, 74.7]	29.0 [5.5, 81.4]	3.6 [2.3, 33.9]	0.219
IL-6 (pg/mL)	12.6 [4.8, 20.3]	9.2 [5.2, 12.9]	17.4 [8.3, 24.3]	0.418
D-dimer (ng/mL)	337 [292, 847]	332 [313, 912]	346 [222, 749]	0.554
**Treatments during hospitalization**				
Antibiotics *n* (%)	12 (44.4)	6 (46.2)	6 (42.9)	0.870
Corticosteroids *n* (%)	5 (18.5)	3 (23.1)	2 (14.3)	0.557
Antiviral drugs *n* (%)	19 (70.4)	10 (76.9)	9 (64.3)	0.472
Immunoglobulin *n* (%)	1 (3.7)	0 (0.0)	1 (7.1)	0.326
Oxygen inhalation *n* (%)	16 (59.3)	9 (69.2)	7 (50.0)	0.310
Mechanical therapy *n* (%)	2 (7.4)	2 (15.4)	0 (0.0)	0.127

*WBC, white blood cell; Hb, hemoglobin; PLT, platelets; ALT, glutamic-pyruvic transaminase; AST, glutamic-oxalacetic transaminease; LDH, lactate dehydrogenase; hs-cTnI, high-sensitivity cardiac troponin I; NT-proBNP, N-terminal pro-B-type natriuretic peptide; hs-CRP, high-sensitivity C-reactive protein; IL-6, interleukin-6*.

### Quality-of-Life Assessments and Exercise Capacity Test

At a 6-month follow-up, none of the patients reported any obvious cardiopulmonary symptoms such as chest distress, chest pain, palpitation, and anhelation. These patients presented normal HR and blood pressure, and there were no significant differences between patients with and without myocardial injury. The SF-36 questionnaire was performed to assess the quality of life of the patients. No significant difference was observed in the SF-36 mean scores for eight specific dimensions between the patients with cardiac injury and those without, as illustrated in Table 2. All patients were also instructed to undergo a 6-min walk test to evaluate the exercise tolerance, except for two with leg pain. The walk distance median was 334 (IQR = 315–358), and oxygen saturation did not decrease after exercise. Although two patients (one in each group) reported obvious fatigue, the blood pressure and dyspnea on the Borg scale after the test did not show any abnormity (Table 2). There was no significant difference in exercise capacity between the patients with and without cardiac injury (Table 2).

**Table 2:** SF-36 questionnaire and 6-min walk test at 6-month follow-up.

**Measurements**	**Overall**	**Patients with cardiac injury**	**Patients without cardiac injury**	***p***
	**(*n* = 27)**	**(*n* = 13)**	**(*n* = 14)**	
**SF-36 questionnaire**				
Physical functioning	85 [73.8, 91.2]	75 [62, 89]	90 [78, 94]	0.193
Role-physical	75 [0, 100]	75 [0, 100]	88 [0, 100]	0.976
Bodily pain	84 [62, 100]	100 [74, 100]	84 [54, 96]	0.161
General health	55 [35, 70]	55 [38, 72]	52 [35, 68]	0.804
Vitality	65 [45, 80]	65 [45, 75]	62 [46, 85]	0.660
Social functioning	63 [25, 63]	62 [50, 75]	62 [25, 62]	0.352
Role-emotional	100 [67, 100]	100 [84, 100]	100 [42, 100]	0.306
Mental health	76 [56, 88]	76 [64, 84]	80 [56, 88]	0.934
Reported health transition	2 [1, 3]	2 [1, 3]	2 [1, 3]	0.908
**6-min walk test**				
Distance (m)	334 [315, 358]	344 [324, 358]	334 [301, 358]	0.624
HR before test (bpm)	75 [68, 85]	75 [69, 88]	75 [67, 84]	0.742
Systolic pressure before test (mmHg)	121 [110, 138]	136 [105, 142]	116 [111, 134]	0.412
Diastolic pressure before test (mmHg)	78 [74, 83]	79 [71, 87]	78 [75, 82]	0.945
Spo_2_ before test (%)	98 [97, 99]	98 [97, 99]	97 [96, 99]	0.505
HR after test (bpm)	75 [68, 85]	75 [69, 88]	75 [67, 84]	0.742
Systolic pressure after test (mmHg)	129 [122, 143]	130 [115, 144]	127 [122, 143]	0.999
Diastolic pressure after test (mmHg)	79 [73, 86]	80 [71, 86]	79 [73, 86]	0.950
Spo_2_ after test (%)	99 [98, 100]	99 [98, 100]	98 [98, 99]	0.356
Borg score after test	0.0 [0.0, 0.4]	0.0 [0.0, 0.0]	0.0 [0.0, 0.5]	0.742

*HR, heart rate*.

### ECG Findings

In patients without cardiac injury, changes of T-wave morphology and ST segment were the most common ECG manifestations (7/14, 50%), especially in older patients. Premature beat (1/14, 7.1%) was also observed. In patients with cardiac injury, ST-segment change (1/13, 7.7%), premature beat (1/13, 7.7%), sinus tachycardia (1/13, 7.7%), sinus bradycardia (1/13, 7.7%), prolongation of PR interval (1/13, 7.7%), and bundle-branch block (2/13, 15.4%) were identified.

### Echocardiography Findings

Echocardiographic characteristics of patients who recovered from COVID-19 are summarized in Table 3. Compared with reference values, no patients showed abnormalities in cardiac structure, as indicated by normal LA dimension, LV dimension, RA dimension, RV dimension, IVS, and LVPW. The functions of LV and RV were preserved, as evidenced by LVEF and TAPSE, respectively. All echocardiographic parameters were statistically comparable between the patients with and without cardiac injury (Table 3).

**Table 3:** Echocardiography, cardiac magnetic resonance imaging and laboratory findings at 6-month follow-up.

**Measurements**	**Overall**	**Patients with cardiac injury**	**Patients without cardiac injury**	***p***
	**(*n* = 27)**	**(*n* = 13)**	**(*n* = 14)**	
**ECHO parameters**				
LA (mm)	34 [30, 38]	31 [30, 34]	35 [34, 38]	0.094
LV (mm)	45 [43, 47]	44 [42, 47]	46 [44, 48]	0.135
RA (mm)	38 [35, 41]	38 [35, 41]	38 [36, 40]	0.897
RV (mm)	25 [21, 29]	23 [20, 29]	26 [22, 29]	0.340
IVS (mm)	10 [9, 11]	10 [9, 10]	11 [9, 11]	0.155
LVPW (mm)	10 [9, 11]	10 [9, 10]	11 [9, 11]	0.134
LVEF (%)	60 [56, 66]	61 [53, 66]	60 [57, 66]	0.700
TAPSE	27 [26, 30]	26 [22, 29]	27 [26, 30]	0.187
**CMR parameters**				
LGE *n* (%)	8 (29.6)	7 (53.8)	1 (7.1)	0.013
LVEF (%)	56 [54, 59]	55 [53, 58]	57 [54, 59]	0.537
EDV (mL)	94 [89, 106]	93 [88, 100]	95 [90, 109]	0.498
ESV (mL)	43 [37, 49]	42 [34, 46]	43 [39, 50]	0.878
Myo mass (g)	65 [57, 73]	63 [51, 70]	68 [60, 75]	0.230
CO (L/min)	3.5 [3.1, 3.9]	3.2 [2.7, 3.6]	3.8 [3.3, 3.9]	0.196
Cl (L/min/m^2^)	2.1 [1.8, 2.3]	2.1 [1.8, 2.3]	2.0 [1.8, 2.3]	0.805
EDVI (mL/m^2^)	57 [52, 63]	60 [54, 66]	54 [50, 61]	0.206
ESVI (mL/m^2^)	25 [22, 31]	26 [23, 31]	24 [22, 29]	0.422
SVI (mL/m^2^)	33 [29, 37]	33 [29, 38]	32 [28, 34]	0.371
Myo mass index (g/m^2^)	38 [35, 43]	40 [33, 43]	38 [35, 42]	0.975
T1 mapping	1211.7 [1185.2, 1247.1]	1242.6 [1202.6, 1265.3]	1205.1 [1179.0, 1236.5]	0.196
ECV	0.28 [0.26, 0.31]	0.28 [0.25, 0.30]	0.29 [0.27, 0.31]	0.618
**Laboratory findings**				
ALT (U/L)	20 [13, 29]	23 [16, 36]	16 [12, 28]	0.152
AST (U/L)	22 [16, 29]	29 [19, 30]	17 [14, 24]	0.004
LDH (U/L)	198 [164, 218]	217 [212, 224]	168 [151, 187]	0.001
Creatinine (umol/L)	58 [50, 68]	65 [57, 78]	52 [47, 60]	0.016
hs-cTnI (pg/mL)	2.9 [2.0, 4.4]	4.3 [2.0, 6.7]	2.5 [1.8, 2.9]	0.041
NT-proBNP (pg/mL)	73 [49, 100]	99 [78, 138]	47 [36, 61]	0.004
hs-CRP (mg/L)	0.9 [0.6, 2.1]	0.8 [0.5, 2.0]	1.5 [0.8, 2.2]	0.356
IL-6 (pg/mL)	1.5 [1.5, 1.9]	1.5 [1.5, 1.5]	1.7 [1.5, 3.0]	0.117
D-dimer (ng/mL)	146 [103, 230]	152 [138, 213]	120 [90, 230]	0.227
ST2 (ng/mL)	14.1 [9.8, 17.9]	16.6 [12.1, 22.5]	12.5 [9.5, 16.7]	0.044

*ECHO, echocardiography; CMR, Cardiac Magnetic Resonance; LA, left atrium; LV, left ventricle; RA, right atrium; RV, right ventricle; IVS, interventricular septum; LVPW, left ventricular posterior wall; SV, stroke volume; LGE, late gadolinium enhancement; LVEF, left ventricular ejection fraction; TAPSE, tricuspid annular plane systolic excursion; EDV, end-diastolic volume; ESV, end-systolic volume; CO, cardiac output; CI, cardiac index; EDVI, end-diastolic volume index; ESVI, end-systolic volume index; SVI, stroke volume index; ECV, extracellular volume; ALT, glutamic-pyruvic transaminase; AST, glutamic-oxalacetic transaminease; LDH, lactate dehydrogenase; hs-cTnI, high-sensitivity cardiac troponin I; NT-proBNP, N-terminal pro-B-type natriuretic peptide; hs-CRP, high-sensitivity C-reactive protein; IL-6, interleukin-6*.

### CMR Findings

**Figure 2:**
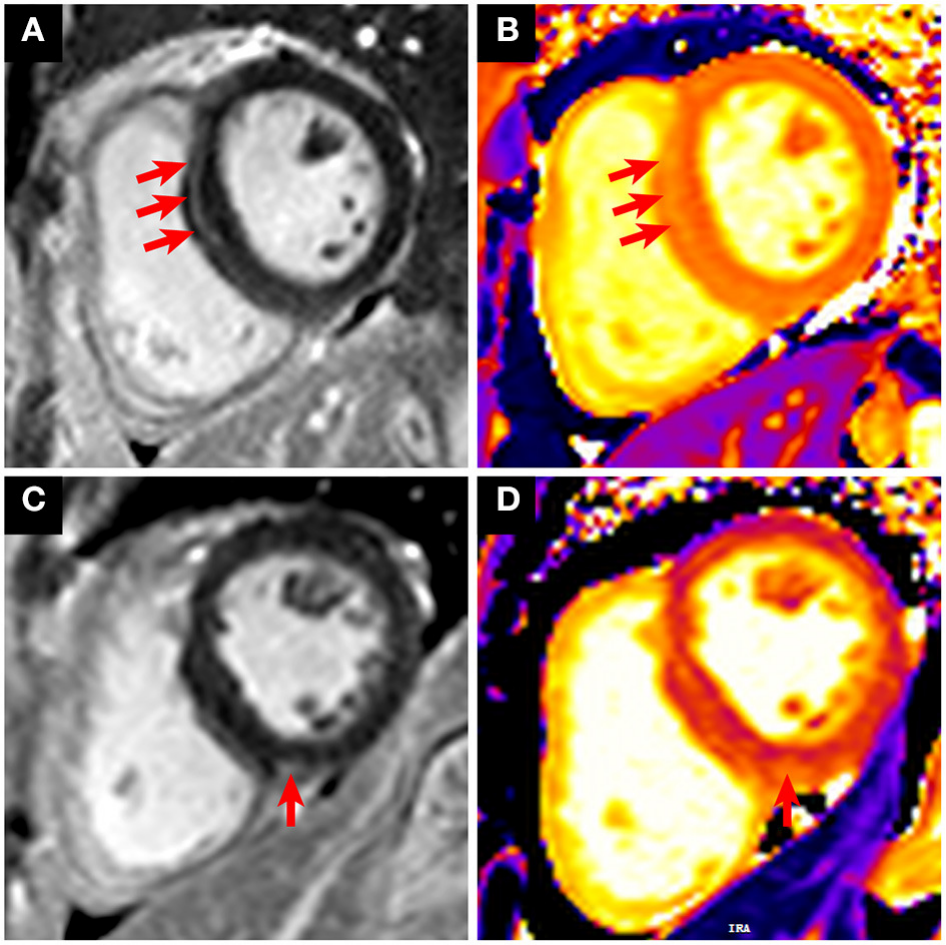
Representative CMR images with LGE positive in patients who recovered from COVID-19. **(A,B)** A 31-year-old male patient with cardiac injury underwent CMR 6 months after recovery from COVID-19. The short-axis LGE sequence showed enhancement in the LV septal segment (**A**, red arrows). Increased native T1 was shown in the corresponding location of focal LGE (**B**, red arrows). **(C,D)** A 63-year-old female patient with cardiac injury underwent CMR 6 months after recovery from COVID-19. The short-axis LGE sequence showed enhancement in the right ventricular insertion point (**C**, red arrow). Increased native T1 was shown in the corresponding location of focal LGE (**D**, red arrow).

Most of the morphological and functional parameters were within the reference range, as indicated by LVEF, cardiac output, cardiac index, end-diastolic volume index, end- systolic volume index, stroke volume index, and myocardial mass index. There were no significant differences among these parameters between the two groups (Table 3). A total of eight patients (8/27, 29.6%) were observed with positive LGE, indicating the existence of myocardial fibrosis. The median of the LGE volume to the total LV myocardium volume ratio was 8.4% (IQR = 7.2%−9.2%; range from 5.5 to 9.9%). Most LGEs (7/8, 87.5%) were located at LV septal segments, followed by RV insertion points (4/8, 50%). Importantly, compared with patients without cardiac injury, patients with cardiac injury exhibited a much higher positive proportion in LGE sequences [7/13 (53.8%) vs. 1/14 (7.1%), *p* = 0.013]. Representative CMR images with LGE positive are shown in Figure 2. There were no significant differences for native T1 and ECV measurements between the two groups.

### Laboratory Findings

Laboratory findings are presented in Table 3. Most of the serum biochemical indexes were within the normal range for recovered patients at the time of 6-month follow-up. However, patients with and without cardiac injury still differed significantly concerning multiple indexes of organ function including the liver, kidney, and heart. Patients with cardiac injury showed significantly higher levels of AST, LDH, creatinine, hs-cTnI, and NT-proBNP, compared with patients without cardiac injury. Notably, consistent with CMR findings, ST2, a recommended indicator of myocardial fibrosis, was higher in patients with cardiac injury (19, 20). No significant differences were observed in the levels of inflammatory factors, interleukin 6, and high-sensitivity C-reactive protein (hs-CRP). The levels of coagulation index d-dimer were also comparable between the two groups.

### Comparison Between Patients Detected With and Without Positive LGE

According to the presence or absence of positive LGE, patients with cardiac injury were further divided into two subgroups (Table 4). Patients in the LGE-positive group were diagnosed with more severe and critical types (6/7 vs. 2/6; Table 4), and more patients were subjected to oxygen inhalation (7/7 vs. 2/6; Table 4) than those with negative LGE. Although the number of patients was limited, compared with the LGE-negative patients, patients with positive LGE presented higher cardiac biomarker (hs-cTnI, median [IQR]: 20.6 [12.6, 27.8] vs. 3.6 [2.8, 4.3], *p* = 0.011), and inflammatory factor (hs-CRP, median [IQR]: 84.2 [73.1, 159.8] vs. 5.2 [1.8, 6.3], *p* = 0.009) on admission. There were no significant differences in these indexes at the 6-month follow-up between the two groups.

**Table 4:** Comparison between patients with and without positive LGE.

**Characteristics**	**Patients with positive LGE**	**Patients without positive LGE**	***p***
	**(*n* = 7)**	**(*n* = 6)**	
Age (years)	63 [62, 73]	61 [58, 65]	0.352
Male *n* (%)	3 (42.9)	1 (16.7)	0.559
Illness classification *n* (%)			0.103
Mild	1 (14.3)	4 (66.7)	
Severe/critical	6 (85.7)	2 (33.3)	
Length of hospital stay (days)	11 [11, 19]	16 [8, 24]	0.667
**Presence of comorbidities**			
History of hypertension *n* (%)	0 (0.0)	1 (16.7)	0.462
History of diabetes mellitus *n* (%)	2 (28.6)	0 (0.0)	0.462
History of coronary heart disease *n* (%)	0 (0.0)	0 (0.0)	-
History of heart failure *n* (%)	0 (0.0)	0 (0.0)	-
History of arrhythmias *n* (%)	0 (0.0)	0 (0.0)	-
History of cardiomyopathy *n* (%)	0 (0.0)	0 (0.0)	-
**Laboratory values on admission**			
WBC (10^9^/L)	4.9 [4.4, 7.2]	6.6 [6.1, 7.8]	0.372
Hb (10^12^/L)	122 [112, 128]	124 [115, 130]	0.685
PLT (10^9^/L)	187 [138, 237]	196 [187, 229]	0.515
ALT (U/L)	32 [28, 45]	33 [23, 35]	0.685
AST (U/L)	34 [28, 48]	29 [19, 46]	0.515
LDH (U/L)	377 [303, 422]	246 [208, 335]	0.465
Creatinine (umol/L)	69 [64, 81]	52 [47, 53]	0.062
hs-cTnI (pg/mL)	20.6 [12.6, 27.8]	3.6 [1.9, 5.6]	0.011
NT-proBNP (pg/mL)	492 [390, 820]	252 [197, 308]	0.165
hs-CRP (mg/L)	84.2 [73.1, 159.8]	5.2 [1.8, 6.3]	0.009
IL-6 (pg/mL)	12.6 [11.3, 14.1]	5.6 [4.8, 6.3]	0.180
D-dimer (ng/mL)	339 [312, 955]	326 [314, 708]	0.685
**Treatments during hospitalization**			
Antibiotics *n* (%)	4 (57.1)	2 (33.3)	0.592
Corticosteroids *n* (%)	2 (28.6)	1 (16.7)	>0.999
Antiviral drugs *n* (%)	5 (71.4)	5 (83.3)	>0.999
Immunoglobulin *n* (%)	0 (0.0)	0 (0.0)	-
Oxygen inhalation *n* (%)	7 (100.0)	2 (33.3)	0.009
Mechanical therapy *n* (%)	1 (14.3)	1 (16.7)	0.906
**ECHO parameters at 6-month follow-up**			
LA (mm)	33 [31, 37]	30 [29, 31]	0.113
LV (mm)	44 [44, 47]	43 [41, 46]	0.385
RA (mm)	40 [37, 42]	35 [34, 37]	0.151
RV (mm)	29 [22, 30]	21 [19, 27]	0.195
IVS (mm)	10 [10, 11]	10 [9, 10]	0.289
LVPW (mm)	10 [10, 10]	10 [9, 10]	0.512
LVEF (%)	61 [54, 65]	60 [54, 66]	0.721
TAPSE	26 [24, 30]	25 [22, 29]	0.886
**CMR parameters at 6-month follow-up**			
LVEF (%)	53 [51, 55]	58 [56, 62]	0.059
EDV (mL)	92 [88, 104]	96 [88, 100]	0.85
ESV (mL)	45 [38, 52]	36 [31, 42]	0.257
Myo mass (g)	67 [62, 83]	48 [42, 56]	0.059
CO (L/min)	3.2 [2.6, 3.7]	3.5 [3.3, 3.6]	0.570
Cl (L/min/m^2^)	1.9 [1.7, 2.2]	2.3 [2.1, 2.4]	0.257
EDVI (mL/m^2^)	55 [54, 62]	64 [59, 68]	0.449
ESVI (ml/m^2^)	26 [25, 32]	26 [21, 30]	0.506
SVI (mL/m^2^)	29 [29, 35]	38 [36, 40]	0.107
Myo mass index (g/m^2^)	40 [37, 49]	33 [30, 38]	0.086
T1 mapping	1248.5 [1243.8, 1287.0]	1202.6 [1192.5, 1215.1]	0.089
ECV	0.28 [0.27, 0.34]	0.27 [0.24, 0.29]	0.562
**Laboratory findings at 6-month follow-up**			
ALT (U/L)	23 [21, 34]	21 [16, 34]	0.774
AST (U/L)	29 [27, 30]	24 [17, 40]	0.566
LDH (U/L)	212 [212, 231]	218 [196, 223]	0.829
Creatinine (umol/L)	70 [61, 81]	61 [53, 69]	0.199
hs-cTnI (pg/mL)	5.7 [3.8, 9.4]	2.8 [1.9, 4.3]	0.317
NT-proBNP (pg/mL)	92 [84, 131]	100 [73, 136]	0.855
hs-CRP (mg/L)	0.8 [0.5, 4.5]	0.7 [0.5, 0.9]	0.391
IL-6 (pg/mL)	1.5 [1.5, 1.5]	1.5 [1.5, 1.5]	0.562
D-dimer (ng/mL)	171 [144, 290]	146 [135, 163]	0.423
ST2 (ng/mL)	14.1 [10.5, 21.2]	18.5 [16.3, 22.0]	0.475

*LA, left atrium; LV, left ventricle; RA, right atrium; RV, right ventricle; IVS, interventricular septum; LVPW, left ventricular posterior wall; SV, stroke volume; LVEF, left ventricular ejection fraction; TAPSE, tricuspid annular plane systolic excursion; EDV, end-diastolic volume; ESV, end-systolic volume; CO, cardiac output; CI, cardiac index; EDVI, end-diastolic volume index; ESVI, end-systolic volume index; SVI, stroke volume index; LGE, late gadolinium enhanced; ECV, extracellular volume fraction; WBC, white blood cell; Hb, hemoglobin; PLT, platelets; ALT, glutamic-pyruvic transaminase; AST, glutamic-oxalacetic transaminease; LDH, lactate dehydrogenase; hs-cTnI, high-sensitivity cardiac troponin I; NT-proBNP, N-terminal pro-B-type natriuretic peptide; hs-CRP, high-sensitivity C-reactive protein; IL-6, interleukin-6*.

## Discussion

To the best of our knowledge, this is the first prospective study reporting 6-month follow-up data of patients who recovered from COVID-19. Of the 27 patients enrolled, no patients reported any obvious cardiopulmonary symptoms at the 6-month follow-up, and there were no statistically significant differences in terms of the quality of life and exercise capacity between the patients with and without cardiac injury, as demonstrated by the SF-36 and 6-min walk tests, respectively. Echocardiography and ECG measurements did not exhibit any obvious abnormalities in these 27 patients, after their recovery from COVID-19 for 6 months. However, comparing the groups of patients with and without cardiac injury, a much higher proportion of positive LGE was found in patients with cardiac injury [7 of 13 (53.8%) vs. 1 of 14 (7.1%), *p* = 0.013], accompanied by the elevation of circulating ST2 level, a recommended indicator of myocardial fibrosis. Patients with cardiac injury presented higher levels of AST, LDH, creatinine, hs-cTnI, and NT-proBNP than those without cardiac injury, which suggested that COVID-19 patients with cardiac injury during hospitalization needed a long-term recovery from cardiac events associated with COVID-19. Nevertheless, these indexes were within the normal range for all recovered patients at the 6-month follow-up.

Previous studies revealed that impaired RV function was detected in patients who recovered from COVID-19 who demonstrated cardiac involvement in the early stage (less than 3 months) of recovery (17, 18). In a recent echocardiographic study in patients with COVID-19, 39% showed RV dilatation dysfunction during hospitalization (21). Compared with patients with normal troponin, patients with elevated troponin presented no significant difference in LV function, but they were identified with worse RV function. However, in our study, recovered patients showed both normal functions of LV and RV, without significant difference between the patients with and without myocardial damage. Longer follow-up duration may be a plausible explanation accounting for this discrepancy. The median duration between discharge and echocardiography was as long as 188 days (IQR = 182–210 days) in our current study. The cardiac function had returned to normal at the 6-month follow-up, although these patients may have suffered from cardiac dysfunction during early convalescence.

An important finding of the CMR assessment is that more than half of patients (7/13, 53.8%) in the cardiac injury group were identified with LGE positive, whereas only one patient (1/14, 7.1%) was observed with LGE positive in patients without cardiac injury. LGE imaging is currently recognized as the gold standard for non-invasive assessment of localized myocardial fibrosis (22). Consistent with the CMR findings, patients with cardiac injury were also detected with higher circulating levels of ST2, a recommended indicator of myocardial fibrosis (19, 20). These results suggest a more frequent existence of cardiac fibrosis in patients with cardiac injury during their convalescence from COVID-19. Further analysis indicated that patients with positive LGE exhibited higher troponin and hs-CRP on admission, although these biomarkers did not differ significantly between the LGE-positive and LGE-negative subgroups in the convalescence stage. These findings provided important insights into the association of myocardial injury in hospitalized patients and cardiac involvement during their recovery from COVID-19. Patients with cardiac injury appeared to be more prone to develop cardiac fibrosis after their recovery. Among these patients, it seemed that more attention should be paid to those with relative higher cardiac biomarkers and inflammatory factors on admission in their convalescence phase. Because of the limited number of included patients, correlation analyses were not conducted. Even so, our results may suggest a possible predictive value of cardiac biomarkers and inflammatory factors in cardiac fibrosis in patients who recovered from COVID-19.

It was reported that cardiac remodeling may occur following viral infection-induced myocardial damage (23). Consistent with this, although LVEF was preserved in all patients who recovered from COVID-19, myocardial fibrosis was detected using CMR, especially in those with elevated troponin during hospitalization. Nevertheless, myocardial fibrosis induced by aging and preexisting cardiac conditions cannot be completely excluded. In our cohort, we noted that a 31-year-old male patient without cardiac comorbidities and a family history of heart disease suffered from myocardial damage followed by COVID-19 infection and was identified with cardiac fibrosis at a 6-month follow-up (Figure 2). It is plausible to believe that cardiac fibrosis may occur in patients during the recovery phase due to COVID-19–triggered myocardial damage, which represents a repair process. Detection of cardiac fibrosis may indicate that these patients were in a relatively early stage of cardiac involvement, and whether it would progress to cardiac dysfunction or electrophysiological disturbance is a potential concern. Further follow-up will be valuable to confirm the long-term clinical implication of cardiac fibrosis in these LGE-positive patients who recovered from COVID-19. Moreover, it would be important to evaluate whether positive LGE and high plasmatic concentration of ST2 could be long-term predictors of cardiac outcomes in a large cohort of recovered COVID-19 patients.

It has been demonstrated that 8–12% of unselected COVID-19 cases were identified with cardiac injury (24). Studies from our group and others have demonstrated that cardiac injury is significantly associated with fatal outcomes of hospitalized patients with COVID-19 (7–12). In the present study, our results implied that cardiac fibrosis during convalescence may be a direct consequence of myocardial damage induced by COVID-19. However, the exact pathophysiological mechanisms underlying myocardial injury induced by COVID-19 remain to be fully elucidated. The possible mechanisms include the direct damage to cardiomyocytes of SARS-CoV-2 infection through angiotensin-converting enzyme 2, cytokine storm precipitated by overactivation of the immune response, dysregulation of the renin–angiotensin–aldosterone system, and disturbances of coagulation and microcirculation-induced hypoxia (3, 5, 25). A previous study revealed a critical role of SARS-CoV in transforming growth factor β signaling, which is a predominant regulator of cardiac fibrosis (26). Given the high homology of the two viruses, SARS-CoV-2 may share a similar mechanism for the contribution to cardiac fibrosis. More mechanism studies are needed to investigate the association between myocardial injury during hospitalization and cardiac fibrosis at recovery in COVID-19 patients.

## Limitations

There are several limitations that should be highlighted. First, in the present study, the sample size of enrolled patients is limited, possibly due to the strict criteria of inclusion and exclusion and the early stage of this outbreak. The findings in this report should be interpreted with caution and warrant further large-scale prospective studies to validate. However, patients in our cohort were recruited from the same hospital, which had detailed clinical data and was confirmed with homogeneity of diagnosis and treatment. Second, we did not observe any major cardiovascular events for now, although cardiac fibrosis was manifested in COVID-19 patients with cardiac injury in our medium-term follow-up. Long-term observation is still needed to further investigate the prognosis of those with cardiac injury after the infection of SARS-CoV-2. Third, pulmonary evaluation such as chest computed tomography and lung function test was not performed in the current study. However, all patients enrolled did not report any pulmonary symptoms at follow-up, and no patients showed abnormity of oxygen saturation and respiration after a 6-min walk test. Finally, even if we have accounted for variables associated with the prognosis of patients with COVID-19 as much as possible, undetected factors might still appear.

## Conclusions

In summary, our preliminary follow-up data with a limited number of patients revealed persistent cardiac involvement in 29.6% (8/27) of recovered patients from COVID-19 up to 6 months after discharge. Patients with cardiac injury during hospitalization were more prone to develop cardiac fibrosis during their recovery. Among patients with cardiac injury, it seemed that those with relatively higher cardiac biomarkers and inflammatory factors were more likely to have cardiac involvement in the convalescence phase. More studies are needed to investigate the association between myocardial injury during hospitalization and cardiac fibrosis in recovered patients from COVID-19.

## Data Availability Statement

The original contributions presented in the study are included in the article/supplementary material, further inquiries can be directed to the corresponding author/s.

## Ethics Statement

The studies involving human participants were reviewed and approved by the ethics committees of Zhongnan Hospital of Wuhan University. The patients/participants provided their written informed consent to participate in this study.

## Author Contributions

ZL, HX, and XWa had full access to all of the data in the study and take responsibility for the integrity of the data and the accuracy of the data analysis, and contributed to the conception and design of the work. XWu, K-QD, and CL acquired and analyzed the data. K-QD and CL performed the statistical analysis. ZY, HH, HC, CZ, TH, and FZ acquired the data and provide technical support. K-QD, CL, HX, and ZL drafted the manuscript. HW, XZ, AC, YY, and XWa provided critical revision of the manuscript for important intellectual content. All authors contributed to the article and approved the submitted version.

## Conflict of Interest

The authors declare that the research was conducted in the absence of any commercial or financial relationships that could be construed as a potential conflict of interest.
